# The effect of happiness-based education on women’s success of water pipe smoking cessation and happiness: a quasi-experimental study

**DOI:** 10.1186/s40359-023-01413-x

**Published:** 2023-11-06

**Authors:** Samira Dehkami, Khatereh Rostami, Zahra Khademian

**Affiliations:** 1 Student Research Committee, School of Nursing and Midwifery, Shiraz University of Medical Sciences, Shiraz, Iran; 2 Community Based Psychiatric Care Research Center, Department of Nursing, School of Nursing and Midwifery, Shiraz University of Medical Sciences, Shiraz, Iran

**Keywords:** Education, Happiness, Nurses, Psychological Models, Public Health, Smoking Cessation, Water Pipe Smoking, Women’s Health services

## Abstract

**Background:**

Water pipe smoking by women threatens their health. Therefore, it is necessary to take measures to reduce this unhealthy behavior. This study aimed to determine the effect of happiness-based education on women’s success of water pipe smoking cessation and happiness.

**Methods:**

This quasi-experimental study was conducted on female water pipe smokers in Iran, from September to January 2021. The participants (n = 68) were selected using convenience sampling and assigned to the intervention and control groups (34 subjects per group) by blocked randomization. The intervention group received a happiness-based education for one month (eight sessions) based on the Fordyce happiness program. The control group did not receive any special education. Data were collected using the Water Pipe Dependence Scale and the Oxford Happiness Questionnaire before and two months after the intervention. Data were analyzed using descriptive statistics, Chi-square, Fisher’s exact test, Independent t-test, Mann-Whitney, and Wilcoxon tests by SPSS software version 22.

**Results:**

Two months after the intervention, the mean change in the happiness score was significantly higher in the intervention group (2.32 ± 2.31) than that in the control group (-0.29 ± 1.81) (P < 0.001). Furthermore, the mean change in the score of water pipe dependence was significantly different between the intervention (-1.44 ± 1.4) and control (0.38 ± 0.85) groups (P < 0.001).

**Conclusion:**

The results showed that education based on Fordyce happiness program can increase the success of water pipe smoking cessation and happiness in women. Therefore, it is recommended to use similar interventions in women’s health promotion programs.

## Background

Tobacco use kills eight million people worldwide annually. Among these, seven million and about 1.2 million deaths are secondary to direct tobacco use and smoke exposure, respectively [1]. Although tobacco is commonly used for cigarette smoking in many countries, nowadays, water pipe smoking (WPS) is an important health issue worldwide, particularly in Eastern Mediterranean countries, Turkey and Iran [2–5]. Besides, WPS is more frequent among men than women, though evidence shows that the WPS rate is increasing among women in many countries [2, 6, 7]. Moreover, women have more positive attitudes about and are more dependent on WPS than men [8, 9]. The prevalence of WPS is 46.2%, 7.8%, and 7.9% among women in Lebanon, Jordan, and Palestine, respectively [2]. In Iran, the prevalence of WPS is 13% [10].

The spread and popularity of WPS in the current societies have been caused by several factors such as the misperception of its harmlessness or being less harmful than cigarette smoking, social acceptance, easy access to various tobacco flavors, and the low cost of hookah consumables [6, 9, 11].

Psychological factors are among the other risk factors for the tendency toward WPS. Individuals with depression and those with sadness and lower levels of happiness are more likely to smoke water pipe and less likely to quit [12–15]. In addition, evidence shows that WPS causes happiness and euphoria in people. Therefore, they use water pipe to avoid sadness, forget problems and feel happy [1, 16]. Hence, enhancing the level of happiness may help individuals quit WPS by providing them with the happiness and pleasure that they are searching for. In this regard, a study showed that high levels of optimism had a protective role in people against WPS [17]. Moreover, a positive psychology and mood management intervention was able to increase the success of people who were motivated to quit cigarette smoking [18]. In addition, a short message motivational program was effective in cigarette smoking cessation [19].

As one of the pioneers of happiness-related studies, Fordyce believes that happiness is one of the gifts provided by nature to human and encompasses a wide range of fundamental efforts for survival, to great scientific and artistic achievements. Fordyce considers that the level of people’s happiness can be increased through education [20]. Educational programs based on Fordyce happiness program increased levels of happiness and general self-efficacy of students [21] and reduced depression of mothers of premature infants [22].

A review of previous studies by the present research team showed that no study is available on the effect of a happiness-based interventions on tobacco use cessation. The WPS is relatively prevalent in the southern cities of Iran. and the women’s tendency toward tobacco use is increasing [6, 7]. Therefore, interventions to reduce WPS in women in these areas are necessary. The present study was conducted to determine the effect of happiness-based education on the success of WPS cessation and women’s happiness.

## Methods

### Study design and participants

The present quasi-experimental study was conducted in Gerash, southern Iran, from September to January 2021. The sample size was calculated according to a previous study aimed at determining the effect of group education programs on happiness in women [23]. Therefore, using the following formula (P-value = 0.05, power 80%, mean1 = 35.16, sd1 = 16.96, mean2 = 33.7, sd2 = 66.06, d = mean1-mean2), a sample size of 28 subjects was estimated in each group with a final sample size of 34 participants, taking into account a 15% attrition rate.

$${\text{n}}_{1}={\text{n}}_{2}=\frac{{\left({\text{z}}_{1-\frac{{\upalpha }}{2}}+{\text{z}}_{1-{\upbeta }}\right)}^{2}\left({{\upsigma }}_{1}^{2}+{{\upsigma }}_{2}^{2}\right)}{{\text{d}}^{2}}$$

Sample was selected by the convenience method. In this way, all eligible women who visited the two primary healthcare centers to receive any services except smoking cessation or psychological services were included in the study. Randomization was done by the block method, in which the 17 random blocks with a capacity of four individuals were formed. The order of the blocks was set by random block generator software. Sampling continued until the sample size in each group was completed. Finally, 34 individuals participated in each group. Inclusion criteria were willingness to participate in the study, an age of 18–60 years, literacy, no history of cigarette smoking and drug use, and not suffering from such diseases as cancer, psychological disorders, and known heart diseases. In addition, a happiness score of < 44, traditional tobacco use, and no use of aromatic and industrial tobaccos were among the inclusion criteria. Exclusion criteria were failure to complete the questionnaires (failure to complete 20% of the main questions), failure to participate in the educational program completely, and initiation of WPS cessation treatment in other methods. There was no sample attrition, and finally the data related to 34 people in each group were analyzed.

### Data collection tools

Data collection tools included a general information form, and the Persian versions of Oxford Happiness Inventory (OHI) and Lebanon Water Pipe Dependence Scale-11 (LWDS-11). Data were collected before and two months after the intervention in both groups. A researcher and a trained research assistant collected the data. They were in regular contact with each other during the data collection and were coordinated in this field.

The general information form consisted of questions about age, marital status, education, employment status, underlying disease, using medication, age of the onset of WPS, frequency of WPS per day, and other questions about WPS status.

Developed by Argyle, Martin, and Crossland (1989), the OHI consists of 29 items each containing four phrases [24]. It is scored as 0 (strongly disagree), 1 (disagree), 2 (agree), and 3 (strongly agree). A final OHI score between 0 and 87 is obtained by the participants, with a higher score indicating more happiness [24, 25]. In a cross-cultural study on students in the UK, Australia, USA and Canada, Francis et al. confirmed its construct validity and reported an alpha coefficient of 0.89–0.99 for the English version of OHI [26]. Liaghatdar et al. confirmed face, content, and construct validity of the Persian version of the inventory based on expert opinions and factor analysis. They reported a Cronbach’s alpha of 0.92 and a test-retest reliability coefficient of 0.73 for the inventory [25].

The LWDS-11 was designed by Salameh et al. (2008) and contains 11 questions in which the first four multiple-choice questions concern the level, duration, and frequency of use as well as the cost of WPS. The choices of the four questions are scored as 0 (never), once (1), sometimes (2), and always (3). Other questions are about the psychological effects of WPS and are scored between 0 and 3. The total scores of this questionnaire range from zero to 33 and a higher score indicates more dependence on WPS. Salameh et al. confirmed face, convergent and discriminative validity of the LWDS-11, and approved its reliability by a Cronbach’s alpha of 0.83 [27]. Pahlavanzadeh et al. confirmed the validity of the Persian version of LWDS-11 using construct validity, and the internal consistency with a Cronbach’s alpha of 0.825. They also reported test-retest reliability of 0.925 [28].

### Intervention

The intervention included a happiness-based education based on the Fordyce happiness program. Fordyce introduced a program consisting of 14 fundamentals. These fundamentals were 14 typical characteristics of happy people that he believed average people can acquire. These fundamentals are: a-being busy and more active; b-spending more time socializing; c-being productive in a meaningful work; d- better organizing and planning for everything; e-stopping worrying; f-reducing expectations and one’s wishes; g-developing positive optimistic thinking; h- being focused on the present time; i-working on a healthy personality; j-creating an outgoing social personality; k-to be oneself; l-eliminating negative feelings and problems; m-to put happiness as the most important priority of oneself; and n-noting that close relationships are the most important sources of happiness [20, 29, 30]. We designed the educational content based on what Fordyce recommended for promoting happiness. We also included cognitive and behavioral techniques to achieve each of the fundamentals. The sessions were held two days a week at 6 pm, agreed upon by the majority of the participants. The content of the sessions is presented in Table 1. The intervention was conducted by the first author (nurse) and a consultant psychologist. Due to the COVID-19 pandemic, these sessions were run online using WhatsApp software. During the first 15 min of each session, the members communicated with each other and discussed the educational issues raised in the previous session, and the participants’ questions were answered. Then, 45 min was devoted to teaching the main topics. During the intervention, educational materials in the form of voice, text, videos, and photos were provided to the intervention group through the WhatsApp social network. Participants were then asked to complete the specified exercises and assignments during the week and to provide feedback in the next session. Participants were provided access to the researcher by telephone to resolve any issues and questions that might arise for them. The control group received no special intervention from the research team.

**Table 1:** Contents of happiness-based education sessions

Session	Content	Assignments
1	Familiarization with the session members, introduction of the program, teaching the goal setting technique and its implementation	Writing a list of daily enjoyable activities, and short-term and long-term goals
2	Introduction of happiness formula, general introduction of happiness techniques, presentation of techniques for increasing physical activity	Starting a new sport or activity Doing good work and reporting it in the group
3	Presenting the happiness formula and a technique for increasing social skills	Practicing the greeting and laughing technique and presenting the results to the group members
4	Teaching the feelings expression technique, developing optimism and positive thinking, avoiding worrying thoughts and prioritizing happiness	Recording imagination about positive activities in the coming future or positive events in the past Doing the thought stopping technique to reduce negative thoughts
5	Social problem-solving, creativity in establishing relationships, and describing its effects in the form of examples Providing a technique for increasing social problem-solving skills and creativity in establishing relationships with oneself and others.	Presenting a report on the creation of new social relationships and its effect on happiness
6	Avoiding worry, teaching a technique for reducing expectations, and a technique for being grateful with words and actions	Recording concerns in a notebook and reviewing it after two weeks
7	Teaching a technique for increasing social intimacy and being oneself, providing a technique for focusing on the present and living in the present	Mindfulness exercises (e.g., meditation) to focus on the present and doing simple and normal tasks to accustom the mind to being in the present
8	Working on increasing physical activities, providing planning techniques, providing happiness prioritization techniques, and reviewing happiness techniques related to previous sessions.	Starting a new sport or activity Preparing a list of tasks in order of priority (weekly schedule)

### Data analysis

Data were analyzed using SPSS software version 22. Descriptive tests were used to describe the data. Qualitative variables between the intervention and control groups were compared using the Chi-square test and Fisher’s exact test. Mean differences of water pipe dependence scores (P = 0.002), mean water pipe dependence scores before (p = 0.02) and after (p < 0.001) the intervention, and happiness scores before intervention (P = 0.009) did not have a normal distribution. The before-after, between-group and within-group variables were compared by the independent t-test or the Mann-Whitney test and the Wilcoxon test, respectively. The Cohen’s d effect size was calculated to evaluate the clinical significance of the findings. The effect sizes of 0.8, 0.5, and 0.2 were considered large, medium, and small, respectively [31]. P < 0.05 was considered a statistically significant level.

## Results

The mean age of participants was 38.79 ± 11.67 years, with mean ages of 36.41 ± 10.12 and 41.18 ± 12.75 in the intervention and control groups, respectively (P = 0.106). The two groups were not statistically different in terms of demographic variables and water pipe use status (Tables 2 and 3, and 4).

**Table 2:** Comparison of demographic variables between intervention and control groups

Variable		Intervention group		Control group		Total		Test statistic	P-value
		**N**	**%**	**N**	**%**	**N**	**%**		
Marital status*	Single	7	20.6	7	20.6	14	20.6	0.324	1.00
	Married	24	70.6	25	73.5	49	72.1		
	Widowed	3	8.8	2	5.9	5	7.4		
	Total	34	100	34	100	68	100		
Occupation*	Employee	3	8.8	4	11.8	7	10.3	3.176	0.428
	Workman	5	14.7	1	2.9	6	8.8		
	Housekeeper	25	73.5	27	79.4	52	76.5		
	Self-employed	1	2.9	2	5.9	3	4.4		
	Total	34	100	34	100	34	100		
Living condition*	Single	1	2.9	0	0	1	1.5	2.143	0.820
	With spouse and children	21	61.8	21	61.8	42	61.8		
	With parents	5	14.7	7	20.6	12	17.6		
	With spouse	3	8.8	4	11.8	7	10.3		
	With children	4	11.8	2	5.9	6	8.8		
	Total	34	100	34	100	68	100		
Level of Education**	Elementary	6	17.6	6	17.6	12	17.6	0.823	0.844
	Secondary school	10	29.4	7	20.6	17	25		
	High school diploma	11	32.4	12	35.3	23	33.8		
	Academic	7	20.6	9	26.5	16	23.5		
	Total	34	100	34	100	68	100		
Underlying disease**	Yes	7	20.6	9	26.5	16	23.5	0.327	0.776
	No	27	79.4	25	73.5	52	76.5		
	Total	32	100	32	100	68	100		
Medication**	Yes	7	20.6	9	26.5	16	23.5	0.327	0.776
	No	27	79.4	25	73.5	52	76.5		
	Total	34	100	34	100	68	100		

*Fisher’s exact test **Chi-square test

**Table 3:** Comparison of water pipe use status (quantitative variables) between intervention and control groups

Variable	Intervention group (n = 34)	Control group (n = 34)	P-value*
	**Mean ± SD**	**Mean ± SD**	
Starting age of water pipe use	28.2 ± 9.69	32.03 ± 9.09	0.101
Years of water pipe use	8.21 ± 9.85	9.15 ± 8.25	0.135
Water pipe use frequency per day	1.50 ± 0.74	1.58 ± 0.78	0.620
Duration of each water pipe use (minutes)	30.74 ± 32.84	26.03 ± 25.22	0.866
Number of tobacco bowls per day	1.59 ± 0.74	1.44 ± 0.66	0.410
Tobacco use level (gram per day)	168.24 ± 51.66	155.88 ± 50.39	0.257

* Mann-Whitney test

The results showed that the intervention and control groups were not statistically different in terms of mean happiness scores before the intervention (P = 0.43). However, a statistically significant difference was observed after the intervention (P = 0.001) so that the post-intervention mean score of happiness was higher in the intervention group than in the control group. In addition, the intragroup mean change in the happiness score was significantly higher in the intervention group (2.32 ± 2.31) than that in the control group (-0.29 ± 1.81) after the intervention (P < 0.001) (Table 5).

According to the results, the mean scores of water pipe dependence between the intervention and control groups were not statistically significant before the intervention (P = 0.814). Two months after the intervention, however, the mean score of water pipe dependence was lower in the intervention group (9.02 ± 1.56) than that in the control group (10.35 ± 2.99) (P = 0.055). The mean change in the score of water pipe dependence was significantly different between the intervention (-1.44 ± 1.4) and control (0.38 ± 0.85) groups (P < 0.001) (Table 5).

**Table 4:** Comparison of water pipe use status (qualitative variables) between intervention and control groups

Variable		Intervention group		Control group		Total		Chi-square test	P-value
		**N**	**%**	**N**	**%**	**N**	**%**		
The reason for Water pipe use*	Entertainment and gathering	15	44.1	13	38.2	28	41.2	1.882	0.889
	Peace of mind	4	11.8	4	11.8	8	11.8		
	Avoid stress	5	14.7	8	23.5	13	19.1		
	Water pipe use by spouse	4	11.8	2	5.9	6	8.8		
	Boredom	4	11.8	4	11.8	8	11.8		
	Escape from loneliness	2	5.9	3	8.8	5	7.4		
	Total	34	100	34	100	68	100		
Water pipe smokers among family members **	Yes	29	85.3	28	82.4	57	83.8	0.108	0.742
	No	5	14.7	6	17.6	11	16.2		
	Total	34	100	34	100	68	100		
Tendency to water pipe cessation**	Yes	26	76.5	26	76.5	52	76.5	0.0	1.00
	No	8	23.5	8	23.5	16	23.5		
	Total	34	100	34	100	68	100		
The reason for the tendency to water pipe cessation**	Reluctance to cessation	8	23.5	8	23.5	16	23.5	0.923	0.630
	The health	18	52.9	21	61.8	39	57.4		
	Spouse’s opposition	8	23.5	5	14.7	13	19.1		
	Total	34	100	34	100	68	100		
Attempts for water pipe use cessation **	Yes	10	29.4	9	26.5	19	27.9	0.073	0.787
	No	24	70.6	25	73.5	49	72.1		
	Total	34	100	34	100	68	100		
Unsuccessful times of water pipe use cessation *	No attempts	24	70.6	25	73.5	49	72.1	2.844	1.00
	Once	5	14.7	6	17.6	11	16.2		
	Twice	2	5.9	1	2.9	3	4.4		
	Thrice	1	2.9	2	5.9	3	4.4		
	Four times	1	2.9	0	0	1	1.5		
	Five times	1	2.9	0	0	1	1.5		
	Total	34	100	34	100	68	100		

*Fisher’s exact test **Chi-square test

**Table 5:** The comparison of happiness and water pipe dependence between the intervention and control groups

Variable	Intervention group (mean ± SD)	Control group (mean ± SD)	P-value***	Effect size
Happiness before interventions* ^†^	38.14 ± 3.21	38.41 ± 3.57	0.43	0.07
Happiness after interventions**^†^	40.47 ± 3.66	37.11 ± 4.02	0.001	0.87
Changes in the happiness score **^ǂ^	2.32 ± 2.31	-0.29 ± 1.81	< 0.001	1.25
Water pipe dependence before interventions*^§^	10.47 ± 2.25	10.14 ± 3.00	0.814	0.12
Water pipe dependence two months after interventions*^§^	9.02 ± 1.56	10.35 ± 2.99	0.055	0.55
Changes in water pipe dependency score*ǂ	-1.44 ± 1.41	0.38 ± 0.85	< 0.001	1.56

*Mann-Whitney test **Independent t-test ***Wilcoxon’s test † Scores range from 0 to 87. ǂ Mean difference = posttest-pretest § Scores range from 0 to 33

## Discussion

This study aimed to determine the effect of happiness-based education on the success of WPS cessation and the happiness of women. The results indicated that the happiness scores of the intervention group improved significantly after the intervention. In addition, their water pipe dependence reduced. Hence, we can conclude that the intervention has had a significant effect on people’s happiness and success in WPS cessation.

Similar to our findings, the effectiveness of happiness-based interventions on the happiness and mental health of people and different groups has also been shown in previous studies. In an Iranian study, six sessions of education based on Fordyce happiness program increased nurses’ happiness [32]. Besides, the education program based on Fordyce happiness program increased the students’ happiness and their mental health [33]. The population in the current study is different from the population in previous studies because the recent study was conducted on women who were water pipe users and had a happiness score below average.

The current findings also revealed the positive effect of the happiness-based intervention on reducing water pipe dependence. Despite not finding studies of happiness-based interventions for water pipe users, similar findings were reported using some emotion management and positive psychology interventions [18, 34]. For instance, Bränström et al. reported the effect of intervention based on emotion management and positive mood on the success of tobacco use cessation in smokers [18]. Similar to our findings, in a study in the USA, an intervention based on positive psychology for eight weeks increased successful cessation in individuals with a low baseline positive affect score [34]. These studies have been used for the population of cigarette smokers.

Altogether, the present results revealed that the happiness-based intervention improved the happiness and success of WPS cessation in women. Moreover, the high effect sizes of mean differences of changes in happiness and water pipe dependence in the present study indicate the clinical significance of the findings. It is noteworthy that the subjects in the intervention group were water pipe smokers for about 8 years, and 85.3% of these participants had family members who were water pipe smokers. The results of a study showed that people who smoked water pipe for more than 12 months had lower intention to quit compared to people who had a shorter smoking duration [35]. Moreover 32.1% of female water pipe users shared water pipe with their family members [36].Therefore, long-term duration of WPS and having family members who smoke water pipe could act as barriers to WPS cessation in this study. Future studies are recommended to investigate the effect of family-centered interventions on the happiness level of individuals and their success in WPS cessation.

According to our findings, most of the participants were housewives in both groups, and the reason for WPS was mentioned by the majority of participants to be entertainment and gathering together. Therefore, it is recommended to use culture-appropriate training, especially for housewives, about healthy entertainment and filling their leisure time as a suitable solution to reduce their tendency toward water pipe use. Future studies are recommended to assess the effectiveness of such interventions.

### Strengths and limitations

The presentation of the intervention using the group formed in the social network is one of the strengths of this study. Moreover, the intervention was implemented by a nurse in collaboration with a psychologist. We showed that with an intervention based on the Fordyce program and using a feasible and convenient method, a nurse was able to create a positive impact on the participants. These results showed the important role of nurses in improving happiness and quitting water pipe in women at the community level. Therefore, the positive effects of the intervention are promising for more participation of nurses in community health promotion services, particularly in the area of women’s health. Moreover, similar interventions that have been used in the field of emotion management [18] and positive psychology [34] have been used for the population of cigarette smokers. However, the nature of water pipe consumption is different from cigarettes in terms of misconceptions about its harmlessness, social acceptance, and its low cost [5, 8, 9]. Hence, we can say this study is unique in its kind and is one of the few studies that have measured the effectiveness of a happiness-based intervention by a nurse with the help of a psychologist on women who smoke water pipe. In addition, the use of a two-group design and randomization were the other strengths of this study.

A limitation of this study was the possibility of information dissemination between the two groups. To prevent this problem, only one person from each family was included in the study to reduce the possibility of sharing educational materials between members of the intervention and control groups. Furthermore, it is worth noting that participants entered the study gradually and thus did not know the other participants. After random assignment, the people in the intervention group joined a virtual social group, but they and the people in the control group did not know each other.

The other limitation of the study was the short follow-up duration for the effectiveness of interventions. It seems that interventions with long-term follow-up can better show the effects of such an intervention.

## Conclusion

The results obtained in this study demonstrate the effect of the happiness-based intervention on increasing the level of happiness and reducing water pipe dependence among women. Therefore, based on these findings, nurses, psychologists, and other professionals active in the area of tobacco use cessation can utilize the interventions based on Fordyce happiness program to facilitate and accelerate the WPS cessation process. The method and findings of the present study can be used to design happiness-based interventions. These education programs can be included in the tobacco use cessation programs. Future studies are recommended to use a combination of psychological interventions with culture-building programs with a longer follow-up duration.

## Data Availability

Data resources and statistical analysis outputs can be provided by the corresponding author (Zahra Khademian) on reasonable request.

## Abbreviations

LWDS-11: Lebanon Water Pipe Dependence Scale 11
OHI: Oxford Happiness Inventory
SPSS: Statistical Package for the Social Sciences
WPS: Water pipe smoking
